# PB.20: Accuracy of specimen radiograph in determining lesion presence in excised specimens, correlating histological and radiological margins

**DOI:** 10.1186/bcr3520

**Published:** 2013-11-08

**Authors:** L Sundaram, N Hartley, M Hoosein, M Al-Attar, E Denton, L Grosvenor, D Lister, G McDonald, S Kaneri

**Affiliations:** 1 Breast Care Centre, Glenfield Hospital, UHL, Leicester, UK

## Introduction

Radiography of the excised specimen post localisation determines whether the target lesion has been removed [1]. The surgical specimen is oriented and if a margin appears suspicious on imaging, further tissue is removed [2]. An incompletely excised carcinoma adds to further surgical workload, psychological and physical morbidity for patients.

## Methods

All patients with biopsy-proven carcinoma undergoing therapeutic excision over a 16-month period were reviewed. A retrospective review of the mammographic lesion localised, visualisation on specimen radiograph (SR), SR report on adequacy, margins of excision, histology of tumour, margins after WLE and further surgery (including minimum distances on margins) was performed and recorded.

## Results

Of 68 therapeutic wire-guided excisions, lesion was accurately detected in SR in 67/68 cases (99%), comparable with the published literature. In 53/68, no more excision was recommended on imaging. Of these, histology confirmed clear margins in 45/68. Further immediate excision was correctly advised in 15 patients. New excision margins were clear in 10/15 cases, thus avoiding second surgery for this subgroup. Extensive disease was seen despite re-excision in five patients. Accuracy of commenting on tumour at margins was 60/68 (88%).

## Conclusion

Discrepancy between radiological and histological margins has significant implications. Accurate SR interpretation is vital. Meticulous attention to the protocol will reduce errors.
